# Supplementary material for: Predicting affinity ties in a surname network
Source: PLoS One. 2021 Sep 2;16(9):e0256603. doi: 10.1371/journal.pone.0256603 (PMC8412287; doi:10.1371/journal.pone.0256603)
Supplement: S1 Table — The results show the performance of the models in hits@{1,3,10}, and Mean Reciprocal Rank (MRR). (PDF) [file pone.0256603.s001.pdf]

**S1 Table. Link prediction for SA19k. The results show the performance of the models in hits@{1,3,10}, and Mean Reciprocal Rank (MRR).**

| Model | H@1 | H@3 | H@10 | MRR |
|---|---|---|---|---|
| TransE | 0.073 | 0.358 | 0.654 | 0.263 |
| TransH | 0.068 | 0.275 | 0.497 | 0.213 |
| DistMult | 0.061 | 0.115 | 0.223 | 0.115 |
| ComplEx | 0.085 | 0.136 | 0.226 | 0.134 |
| ConvE | 0.193 | 0.381 | 0.593 | 0.325 |
| RotatE | 0.181 | 0.388 | **0.668** | 0.332 |
| CrossE | 0.153 | 0.351 | 0.610 | 0.229 |
| ConvTransE | 0.171 | 0.324 | 0.488 | 0.278 |
| SACN | 0.156 | 0.275 | 0.408 | 0.243 |
| TuckER | **0.214** | **0.406** | 0.607 | **0.346** |
